# Supplementary figures and images for: A retrospective study using machine learning to develop predictive model to identify rotavirus-associated acute gastroenteritis in children
Source: PeerJ. 2025 Apr 14;13:e19025. doi: 10.7717/peerj.19025 (PMC12005185; doi:10.7717/peerj.19025)

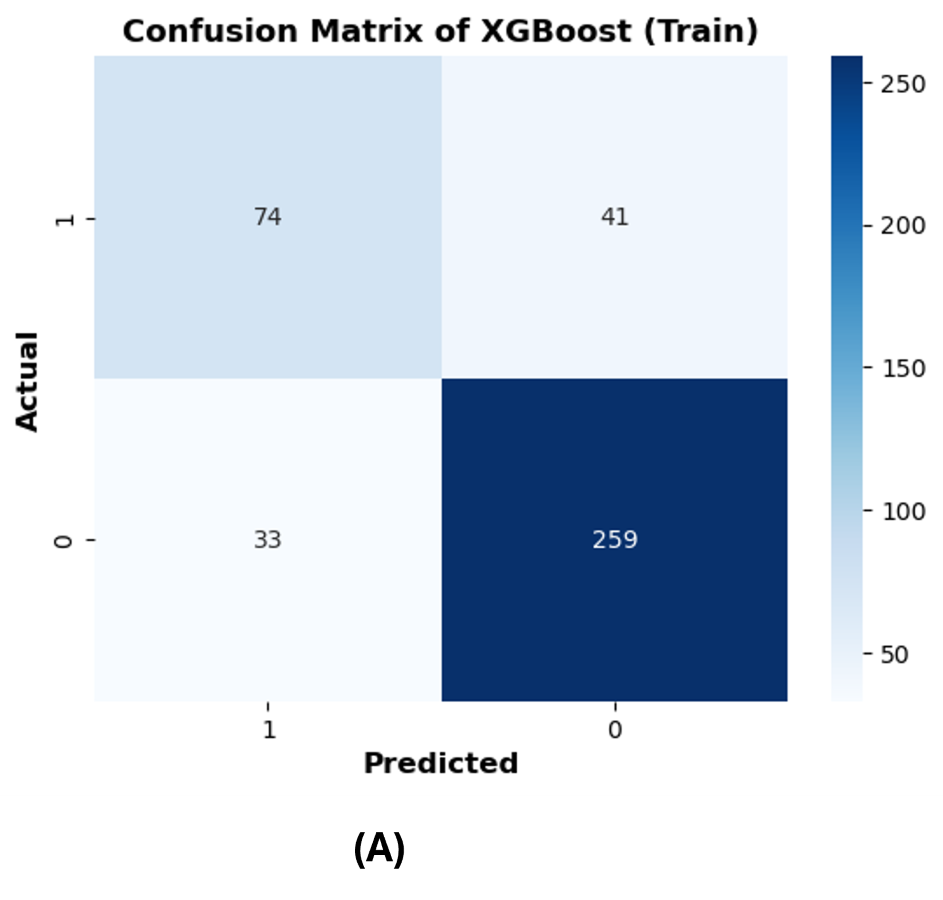

Supplement: Supplemental Information 1 — (A) train dataset of XGBoost. [file peerj-13-19025-s001.png]

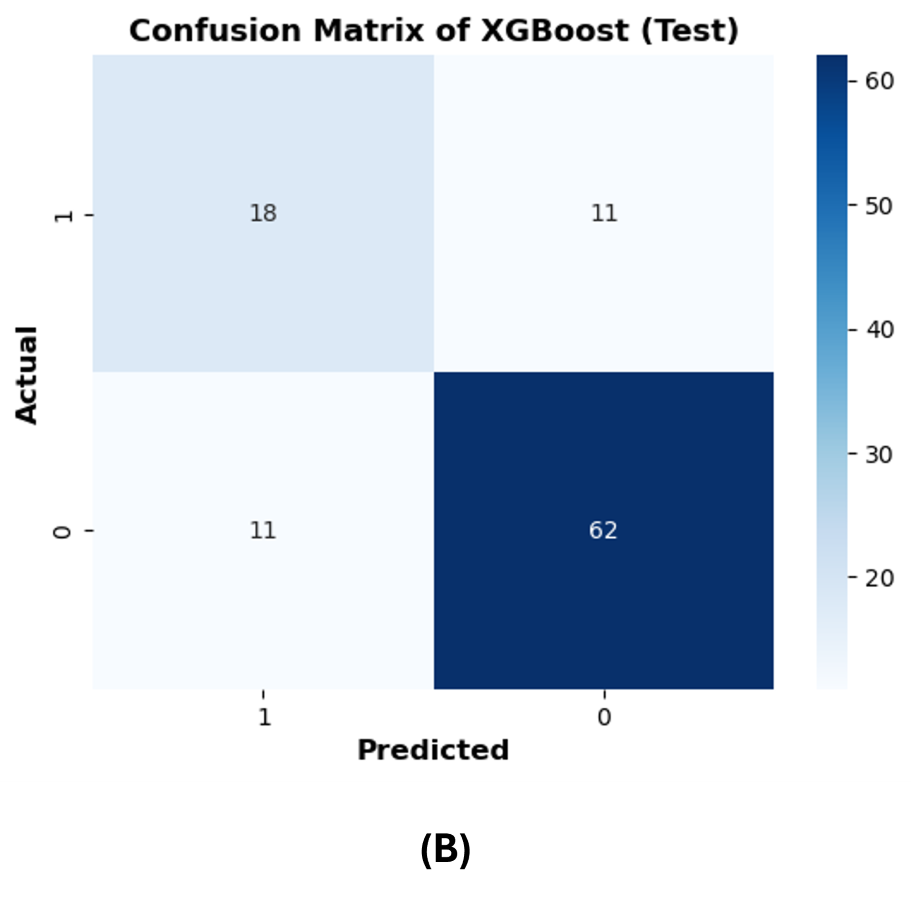

Supplement: Supplemental Information 2 — (B) test dataset of XGBoost. [file peerj-13-19025-s002.png]

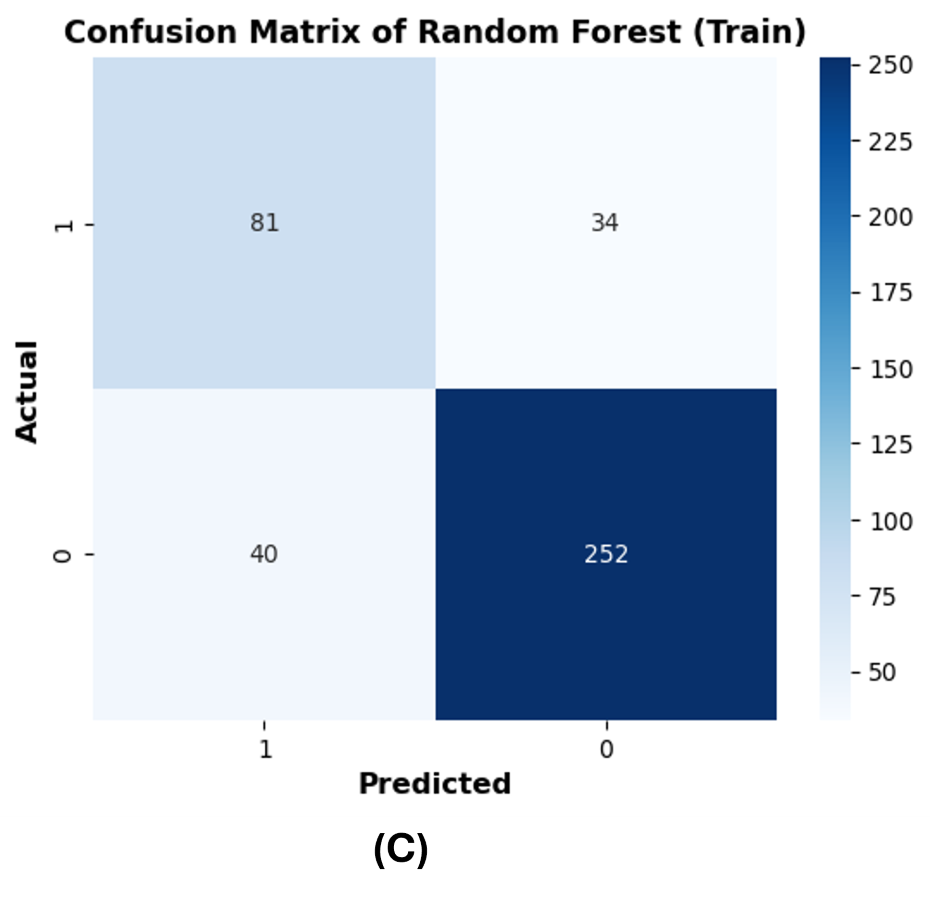

Supplement: Supplemental Information 3 — (C) train dataset of Random Forest. [file peerj-13-19025-s003.png]

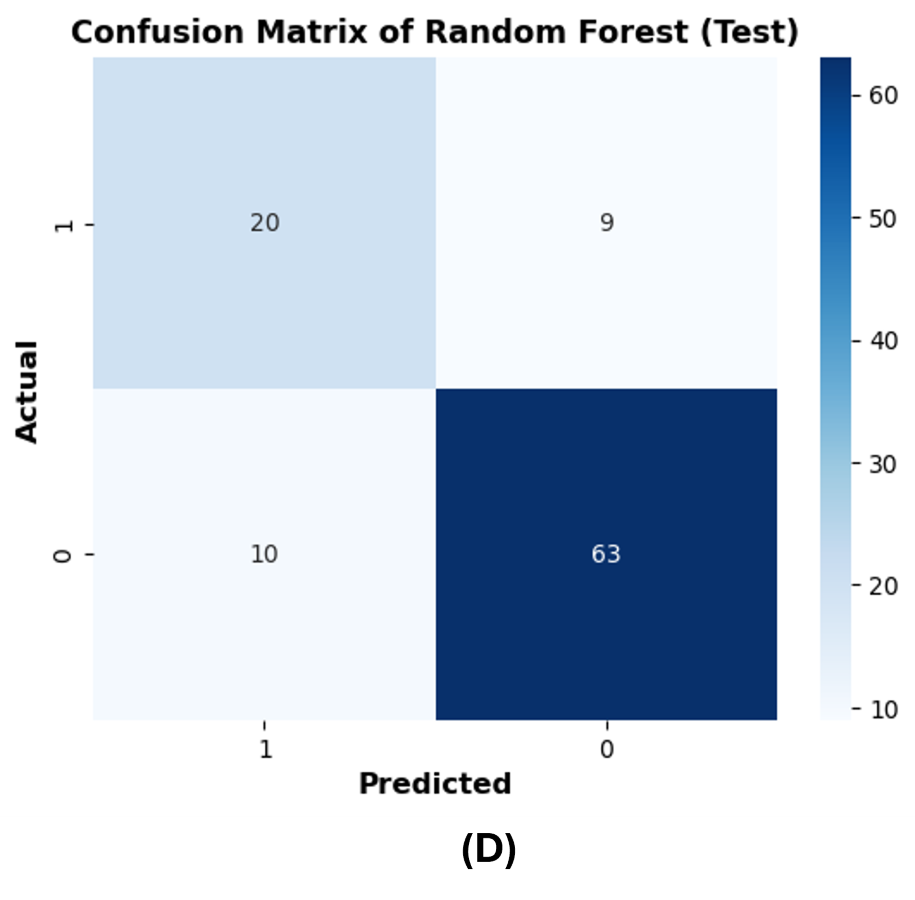

Supplement: Supplemental Information 4 — (D) test dataset of Random Forest. [file peerj-13-19025-s004.png]

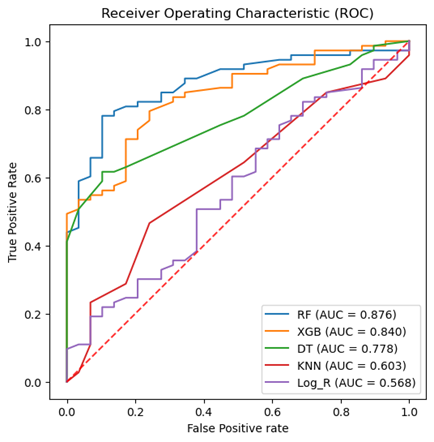

Supplement: Supplemental Information 5 — Five supervised ML algorithms (RF, XGB, DT, KNN, & Log_R) showed good performance based on the receiver operator characteristic curve (ROC). The ROC curve takes the false-positive rate as the horizontal axis and the true-positive rate as the vertical axis. The horizontal axis represents the proportion of the actual negative instances in the positive class predicted by the classifier to all negative instances. The vertical axis represents the proportion of the actual positive instances in the positive class predicted by the classifier to all positive instances. The area under the curve (AUC) represents the ability of models to differentiate between positive and negative values during prediction. [file peerj-13-19025-s005.png]

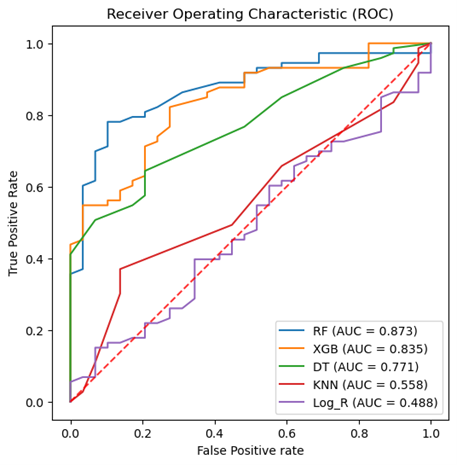

Supplement: Supplemental Information 6 — Five supervised ML algorithms (RF, XGB, DT, KNN, & Log_R) showed good performance based on the receiver operator characteristic curve (ROC). The ROC curve takes the false-positive rate as the horizontal axis and the true-positive rate as the vertical axis. The horizontal axis represents the proportion of the actual negative instances in the positive class predicted by the classifier to all negative instances. The vertical axis represents the proportion of the actual positive instances in the positive class predicted by the classifier to all positive instances. The area under the curve (AUC) represents the ability of models to differentiate between positive and negative values during prediction. [file peerj-13-19025-s006.png]
