# Supplementary material for: A retrospective study using machine learning to develop predictive model to identify rotavirus-associated acute gastroenteritis in children
Source: PeerJ. 2025 Apr 14;13:e19025. doi: 10.7717/peerj.19025 (PMC12005185; doi:10.7717/peerj.19025)
Supplement: Supplemental Information 7 — The training time for seven supervised machine learning algorithms. [file peerj-13-19025-s007.docx]

| **ML_models** | **CPU time (ms)** | **Wall time (ms)** |
| --- | --- | --- |
| SVM | 78.1 | 65.7 |
| KNN | 78.1 | 104 |
| Log_Reg | 109 | 40.3 |
| RF | 656 | 214 |
| DT | 15.6 | 9.56 |
| XGBoost | 719 | 1.3 |
| NB | 15.6 | 21.7 |
