# Supplementary material for: A retrospective study using machine learning to develop predictive model to identify rotavirus-associated acute gastroenteritis in children
Source: PeerJ. 2025 Apr 14;13:e19025. doi: 10.7717/peerj.19025 (PMC12005185; doi:10.7717/peerj.19025)
Supplement: Supplemental Information 8 — The accuracy of seven supervised machine learning algorithms evaluated in the absence of either fever or temperature. [file peerj-13-19025-s008.docx]

| **Algorithms** | **Accuracy (All data)** | **Accuracy (Without fever)** | **Accuracy**  **(Without temperature)** |
| --- | --- | --- | --- |
| SVM | 71.57 | 71.57 | 71.57 |
| KNN | 69.6 | 68.6 | 71.56 |
| Logistic Regression | 71.6 | 71.56 | 71.56 |
| Naïve Bayes | 65.7 | 69.6 | 64.7 |
| Decision Tree | 72.5 | 72.54 | 72.54 |
| Random Forest | 81.4 | 81.37 | 80.39 |
| XGboost | 78.4 | 78.43 | 77.4 |
