# Supplementary material for: A retrospective study using machine learning to develop predictive model to identify rotavirus-associated acute gastroenteritis in children
Source: PeerJ. 2025 Apr 14;13:e19025. doi: 10.7717/peerj.19025 (PMC12005185; doi:10.7717/peerj.19025)
Supplement: Supplemental Information 9 [file peerj-13-19025-s009.docx]

**Codebook to convert numbers to their respective factors**

The Modified_file_final_Sourav (1) file contains 11 columns, that contains detailed overview of symptoms for 509 different individuals.

| Column | Description | Formula |
| --- | --- | --- |
| Sample labelling in Record Book | Data type: Text  Context: Describes about the id numbers for different individuals |  |
| Vomiting episodes per day | Data type: Numerical  Context: Describes about the time of vomiting episodes per day |  |
| Vomiting duration (days) | Data type: Numerical  Context: Describes about the duration time of vomiting per day |  |
| Diarrhea duration (days) | Data type: Numerical  Context: Describes about the total no of days of diarrhea |  |
| Fever | Data type: Categorical | + represents Positive case of fever  And - represents Negative case |
| Maximum number of stools (days) | Data type: Numerical  Context: Describes about the total no episodes of stools per day |  |
| Dehydration | Data type: Numerical |  |
| Temperature_Mild | Data type: Numerical  Context: Temperature mild or not | 1 represents Mild temperature  And 0 represents abnormal temperature {that may normal or moderate temperature} |
| Temperature_Moderate | Data type: Numerical  Context: Temperature moderate or not | 1 represents Moderate temperature  And 0 represents abnormal temperature {that may normal or mild temperature} |
| Temperature_Normal Data | Data type: Numerical  Context: Temperature normal or not | 1 represents Normal temperature  And 0 represents abnormal temperature |
| Rotavirus (+ve/-ve) by ELISA | Data type: Numerical  Context: Rotaviral infection positive or not | 1 represents Positive case  And 0 represents Negative case |
